# Residential environment in relation to self-report of respiratory and asthma symptoms among primary school children in a high-polluted urban area

**DOI:** 10.1038/s41598-022-06919-9

**Published:** 2022-02-22

**Authors:** Nawarat Apichainan, Saowanee Norkaew, Nutta Taneepanichskul

**Affiliations:** 1 Department of Health, Ministry of Public Health, Nonthaburi, 11000 Thailand; 2 Faculty of Public Health, Thammasat University (Rungsit Campus), Pathumthani, 12121 Thailand; 3 College of Public Health Sciences, Chulalongkorn University, Institute Building 2-3, Phyathai Rd, Pathumwan, Bangkok, 10330 Thailand; 4 HAUS IAQ Research Unit, Department of Pediatrics, Faculty of Medicine, Chulalongkorn University King Chulalongkorn Memorial Hospital, Bangkok, Thailand

**Keywords:** Risk factors, Public health, Signs and symptoms, Respiratory signs and symptoms

## Abstract

Respiratory disease and its complication are the cause of children deaths worldwide every year. Several epidemiological studies pointed out an association between quality of residential in inner city and risk of children health. However, few studies had been focused in high-polluted urban area in low to middle income countries. A cross-sectional study was conducted to investigate the association between residential environments and respiratory symptoms including asthma among 658 primary school children living in urban area of Bangkok Thailand. The International Study of Asthma and Allergies in Childhood (ISAAC) questionnaire was modified to access symptoms during the past 12 months. Binary logistic regression model was performed. Living near garment and clothing shop is associated with shortness of breath (AOR = 1.846; 95% CI 1.034, 3.297). Vectors in home is related to dry cough at night (AOR = 1.505; 95% CI 1.052, 2.153) and phlegm (AOR = 1.414; 95% CI 1.014, 1.973). Wall dampness is increased odd of having wheezing or whistling (asthma) in the chest (AOR = 1.921; 95% CI 1.141, 3.235). Children age, gender, and a family history of asthma were modified the associations. Our finding may provide strategies focusing on living environment improvement with a specific group of children to address respiratory disease prevention.

## Introduction

Exposure to unhealthy environment is predicted to affect 25% of 5.9 million all children global mortality. One third of the death were attributed to respiratory disease and its complications^1^. The prevalence of respiratory disease and asthma in children is increasing in low-to middle-income countries^2^. Children with respiratory disease and asthma were accounted for 64.7% and 29.4% in Thailand, while most of them were found in urban area^3,4^. Asthma symptoms are defined by repeated attacks of wheezing, breathlessness, and dry cough at night, which differ in each affected person^2^. The attacks interrupt daily activities, sleep quality, and school attendance among children^2,5^.

Living in unhealthy environment and exposing to unhealthy air can adversely affect the respiratory and asthma symptoms. Air pollution is possible to activate systematic inflammatory process which impact on oxidative stress. It can induce several pathological conditions including respiratory system^6^. Source of exposure to unhealth environment among children are their home condition, school, and outdoor environment^7^. Housing environments, such as household mold^8^, dampness, pets, vectors (i.e., cockroach and rat), carpets, and curtains have been found to be significant risk factors for respiratory and asthma symptoms^9^. Although asthma exacerbation is caused by children living conditions such as house dust mites, mold, and dampness, the explanation is still unclear^10–12^. Living with pet in the early stages of life leads to respiratory complications, including asthma symptoms, rhino conjunctivitis, and eczema in young adults^13^.

Additionally, tobacco smoke, fuel-burning combustion, and building materials are also recognized as a common source of indoor air pollution.

Children living in high-polluted area are possible to expose to higher indoor pollutants than low-polluted area. A study in India suggested that indoor suspended particulate matter (SPM) of asthmatic children home in inner city was higher than non-asthmatic children^14^. However, few studies have focused on residential environments and respiratory and asthma symptoms in children living in the cities of low-to middle-income countries. Because of the increased understanding of respiratory and asthma symptoms, it is crucial to gain better knowledge of the link between residential environments and the risk of respiratory and asthma symptoms among children living in highly polluted urban areas of Thailand.

## Results

### Children’s characteristics and respiratory and asthma symptoms

A total of 658 primary school children, male (50.2%) and female (49.8%), were included in this study. Table 1 shows that the median age (interquartile range, IQR) was 8 (2) years. The median height of the male and female children was 122 (10.0) cm. The median weight at birth was 3 (0.3) kg, and the weight during this study was 26 (7.0) kg. Most of the children (64.6%) had exercised regularly. Only 2.3% had a family history of asthma. The highest prevalence of respiratory and asthma symptoms in the previous 12 months was running nose without cold symptoms (52.7%) (Supplement Table S1). The children stayed at their residences on weekends more than on school days. Most of the children (53.0%) were in their place of residence for approximately 24 h during the weekend, whereas 76.0% were at home for 13–14 h during the school day. The majority of children (69.5%) spent most of their time in their bedrooms.

**Table 1:** Children characteristics and respiratory symptoms (12 months) (n = 658).

Children characteristics	Total (n = 658)		Wheezing or whistling in the chest (asthma)					Dry cough at night					Phlegm					Shortness of breath					Running nose without cold				
	n	(%)	Yes (n = 75): n (%)		No (n = 583): n (%)		p-value	Yes (n = 214): n (%)		No (n = 444): n (%)		p-value	Yes (n = 285): n (%)		No (n = 373): n (%)		p-value	Yes (n = 60): n (%)		No (n = 598): n (%)		p-value	Yes (n = 347): n (%)		No (n = 311): n (%)		p-value
Age (years); median (IQR)	8	(2.0)	8	(2.0)	8	(2.0)	0.308^a^	8	(2.0)	8	(2.0)	0.129^a^	8	(2.0)	8	(2.0)	0.277^a^	8	(2.0)	8	(2.0)	0.232^a^	8	(2.0)	8	(2.0)	0.725^a^
**Gender**							0.186^b^					0.345^b^					0.212^b^					0.980^b^					0.210^b^
Male	330	(50.2)	43	(13.0)	287	(87.0)		113	(34.2)	217	(65.8)		135	(40.9)	195	(59.1)		30	(9.1)	300	(90.9)		166	(50.3)	164	(49.7)	
Female	328	(49.8)	32	(9.8)	296	(90.2)		101	(30.8)	227	(69.2)		150	(45.7)	178	(54.3)		30	(9.1)	298	(90.9)		181	(55.2)	147	(44.8)	
Height (cm); Median (IQR)	122	(10.0)	123	(10.0)	122	(90.2)	0.815^a^	120	(10.0)	124	(10.0)	0.008^a^	122	(10.0)	122	(10.0)	0.743^a^	120	(10.0)	122	(10.0)	0.648^a^	123	(10.0)	121	(10.0)	0.835^a^
**Weight (kg)**																											
Present (kg); median (IQR)	26	(7.0)	26	(7.0)	26	(7.0)	0.757^a^	26	(8.0)	26	(7.4)	0.164^a^	26	(7.0)	26	(7.0)	0.742^a^	26	(8.4)	26	(7.0)	0.328^a^	26	(7.0)	26	(7.0)	0.737^a^
At birth (kg); median (IQR)	3	(0.3)	3	(0.2)	3	(0.3)	0.330^a^	3	(0.3)	3	(0.3)	0.892^a^	3	(0.3)	3	(0.2)	0.435^a^	3	(0.4)	3	(0.3)	0.003^a^	3	(0.3)	3	(0.2)	0.802^a^
**Family history of asthma**							0.685^c^					0.580^c^					0.065^b^					0.639^c^					0.106^b^
Yes	15	(2.3)	2	(13.3)	13	(86.7)		6	(40.0)	9	(60.0)		10	(66.7)	5	(33.3)		2	(13.3)	13	(86.7)		11	(73.3)	4	(26.7)	
No	643	(97.7)	73	(11.4)	570	(88.6)		208	(32.3)	435	(67.7)		275	(42.8)	368	(57.2)		58	(9.0)	585	(91.0)		336	(52.3)	307	(47.7)	
**Exercise**							0.726^b^					0.090^b^										0.776^b^					0.244^b^
Yes	319	(64.6)	38	(11.9)	281	(88.1)		93	(29.2)	226	(70.8)		130	(40.8)	189	(59.2)	0.152^b^	26	(8.2)	293	(91.8)		163	(51.1)	156	(48.9)	
No	175	(35.4)	19	(10.9)	156	(89.1)		64	(36.6)	111	(63.4)		83	(47.4)	92	(52.6)		13	(7.4)	162	(92.6)		99	(56.6)	76	(43.4)	

^a^Mann–Whitney *U* test, ^b^Pearson Chi-square test, ^c^Fisher's exact test.

### Residential environment and respiratory/asthma symptoms

Table 2 presents the residential characteristics and respiratory symptoms of the primary school children who participated in this study. Most children lived in flats, apartments, or condominiums (62.9%). Average age of house was more than 30 years, which was not associated with respiratory and asthma symptoms. The majority of the participants were tenants (77.1%) rather than owners (22.9%). The type of residence was not associated with any respiratory symptoms. Among the places reported to be near the residence, the highest was garment/clothing factory (24.3%), which was associated with shortness of breath (p = 0.008).

**Table 2:** Residential characteristics and respiratory/asthma symptoms of primary school Children (n = 658).

Residential environment	Total (n = 658)		Wheezing or whistling in the chest (asthma)					Dry cough at night					Phlegm					Shortness of breath					Running nose without cold				
	n (%)		Yes (n = 75): n (%)		No (n = 583): n (%)		p-value	Yes (n = 214): n (%)		No (n = 444): n (%)		p-value	Yes (n = 285): n (%)		No (n = 373): n (%)		p-value	Yes (n = 60): n (%)		No (n = 598): n (%)		p-value	Yes (n = 347): n (%)		No (n = 311): n (%)		p-value
**Type of residence**																											
Single family house	109	(16.6)	12	(11.0)	97	(89.0)	0.282^b^	27	(24.8)	82	(75.2)	0.214^b^	44	(40.4)	65	(59.6)	0.686^b^	6	(5.5)	103	(94.5)	0.507^b^	54	(49.5)	55	(50.5)	0.785^b^
Townhouse	85	(12.9)	15	(17.6)	70	(82.4)		29	(34.1)	56	(65.9)		35	(41.2)	50	(58.8)		8	(9.4)	77	(90.6)		46	(54.1)	39	(45.9)	
Flat/apartment/condominium	414	(62.9)	43	(10.4)	371	(89.6)		138	(33.3)	276	(66.7)		181	(43.7)	233	(56.3)		42	(10.1)	372	(89.9)		218	(52.7)	196	(47.3)	
Community (slum)	50	(7.6)	5	(10.0)	45	(90.0)		20	(40.0)	30	(60.0)		25	(50.0)	25	(50.0)		4	(8.0)	46	(92.0)		29	(58.0)	21	(42.0)	
Age of residence (year); Median (IQR)	30	(10.0)	30	(10.0)	30	(10.0)	0.242^a^	30	(10.0)	30	(10.0)	0.162^a^	30	(12.0)	30	(10.0)	0.179^a^	30	(12.0)	30	(10.0)	0.560^a^	30	(15.0)	30	(10.0)	0.352^a^
**Residence owner**																											
Owner	151	(22.9)	10	(6.6)	141	(93.4)	0.035^b^	44	(29.1)	107	(70.9)	0.312^b^	69	(45.7)	82	(54.3)	0.501^b^	12	(7.9)	139	(92.1)	0.569^b^	81	(53.6)	70	(46.4)	0.799^b^
Tenant	507	(77.1)	65	(12.8)	442	(87.2)		170	(33.5)	337	(66.5)		216	(42.6)	291	(57.4)		48	(9.5)	459	(90.5)		266	(52.5)	241	(47.5)	
**Place near residence**																											
Furniture shop							0.433^c^					0.414^b^					0.712^b^					0.155^c^					0.636^b^
Yes	39	(5.9)	6	(15.4)	33	(84.6)		15	(38.5)	24	(61.5)		18	(46.2)	21	(53.8)		6	(15.4)	33	(84.6)		22	(56.4)	17	(43.6)	
No	619	(94.1)	69	(84.6)	550	(15.4)		199	(61.5)	420	(38.5)		267	(53.8)	352	(46.2)		54	(84.6)	565	(15.4)		325	(43.6)	294	(56.4)	
Garment/clothing							0.724^b^					0.442^b^					0.811^b^					0.008^b^					0.910^b^
Yes	160	(24.3)	17	(10.6)	143	(89.4)		56	(35.0)	104	(65.0)		68	(42.5)	92	(57.5)		23	(14.4)	137	(85.6)		85	(53.1)	75	(46.9)	
No	498	(75.7)	58	(89.4)	440	(10.6)		158	(65.0)	340	(35.0)		217	(57.5)	281	(42.5)		37	(85.6)	461	(14.4)		262	(46.9)	236	(53.1)	
Garage/car care							0.173^b^					0.819^b^					0.734^b^					0.702^b^					0.948^b^
Yes	64	(9.7)	4	(6.3)	60	(93.8)		20	(31.3)	44	(68.8)		29	(45.3)	35	(54.7)		5	(7.8)	59	(92.2)		34	(53.1)	30	(46.9)	
No	594	(90.3)	71	(93.7)	523	(6.2)		194	(68.7)	400	(31.2)		256	(54.7)	338	(45.3)		55	(92.2)	539	(7.8)		313	(46.9)	281	(53.1)	
Petrol station							0.789^c^					0.709^b^					0.739^b^					0.766^c^					0.394^b^
Yes	37	(5.6)	3	(8.1)	34	(91.9)		11	(29.7)	26	(70.3)		17	(45.9)	20	(54.1)		4	(10.8)	33	(89.2)		17	(45.9)	20	(54.1)	
No	621	(94.4)	72	(91.9)	549	(8.1)		203	(70.3)	418	(29.7)		268	(54.1)	353	(45.9)		56	(89.2)	565	(10.8)		330	(54.1)	291	(45.9)	
Fresh market and restaurant							0.398^c^					0.407^c^					0.430^b^					0.384^c^					0.041^b^
Yes	15	(2.3)	0	(0.0)	15	(100.0)		3	(80.0)	12	(20.0)		5	(33.3)	10	(66.7)		0	(0.0)	15	(100.0)		4	(26.7)	11	(73.3)	
No	643	(97.7)	75	(100)	568	(0.0)		211	(20.0)	432	(80.0)		280	(66.7)	363	(33.3)		60	(100.0)	583	(0.0)		343	(73.3)	300	(26.7)	

^a^Mann–Whitney *U* test, ^b^Pearson Chi-square test, ^c^Fisher's exact test.

Children living in an environment of cigarette smoke (p = 0.005) and with smokers in the family (p = 0.024) were associated with a dry cough at night. The presence of vectors was associated with the symptoms of dry cough at night (p = 0.005), phlegm (p = 0.006), and running nose without cold (p = 0.009). The presence of wall dampness in the residence was associated with the symptoms of wheezing or whistling in the chest (asthma) (p = 0.007), phlegm (p = 0.024), and shortness of breath (p = 0.004). Home renovation was associated with the symptoms of dry cough at night (p = 0.029), phlegm (p = 0.028), and running nose (p = 0.021). However, stoves used for cooking in the residence, including charcoal smoke, flowers with pollen, pets, incense, charcoal smoke, incense smoke, and insecticide use, did not show any associations with respiratory and asthma symptoms (Supplement Table S2).

A binary logistic regression model was used to assess the association between residential environments and respiratory and asthma symptoms in the previous 12 months (Table 3). The results showed that compared with the absence of wall dampness, its presence increased by 1.921-fold the likelihood of wheezing or whistling in the chest (asthma) (AOR = 1.921; 95% CI 1.141–3.235; p = 0.014) and by 1.882-fold the likelihood of shortness of breath (AOR = 1.882; 95% CI 1.047–3.386; p = 0.035). Compared with their absence, a garment/clothing factory near the residence increased the likelihood of shortness of breath by 1.846-fold (AOR = 1.846; 95% CI 1.034–3.297; p = 0.038). Compared with the absence of vectors, their presence increased by 1.505-fold the likelihood of having dry cough at night (AOR = 1.505; 95% CI 1.052–2.153; p = 0.025) and 1.414-fold the likelihood of phlegm (AOR = 1.414; 95% CI 1.014–1.973; p = 0.041). However, home renovation and the presence of flowers with pollen were possible risk factors for respiratory and asthma symptoms (AOR > 1) among children, but statistical significance was not achieved.

**Table 3:** Binary logistic regression model of associations between residential environment and respiratory/asthma symptoms (n = 658).

Factors	Wheezing or whistling in the chest (asthma)^a,b^							Dry cough at night^a,b^						Phlegm^a,b^						Shortness of breath^a,b^						Running nose without cold^a,b^				
	AOR (95% CI)					*p*-value		AOR (95% CI)				*p*-value		AOR (95% CI)				*p*-value		AOR (95% CI)				*p*-value		AOR (95% CI)				*p*-value
Age of residence (year)								1.009		(0.995, 1.023)		0.222		1.009		(0.996, 1.022)		0.186												
**Place near residence**																														
Garment/clothing (yes)																				1.846		(1.034, 3.297)		0.038						
Furniture shop (yes)																				1.696		(0.643, 4.473)		0.286						
Garage/car care (yes)	0.501		(0.174, 1.442)			0.200																								
Fresh market and restaurant (cooking smoke) (yes)																										0.283		(0.087, 0.915)		0.035
Living in cigarette smoke area (yes)								1.643		(0.980, 2.755)		0.060		1.254		(0.755, 2.084)		0.383								1.093		(0.646, 1.849)		0.740
Living in incense smoke area (yes)	2.030		(0.700, 5.887)			0.192														3.013		(0.986, 9.207)		0.053		1.741		(0.680, 4.456)		0.248
Vectors (cockroach, rat, etc.) (yes)	1.303		(0.763, 2.224)			0.332		1.505		(1.052, 2.153)		0.025		1.414		(1.014, 1.973)		0.041		1.328		(0.717, 2.461)		0.367		1.382		(0.995, 1.919)		0.054
Home renovation (yes)								1.341		(0.896, 2.007)		0.155		1.294		(0.877, 1.911)		0.194		1.538		(0.822, 2.878)		0.178		1.388		(0.934, 2.062)		0.105
Wall dampness (yes)	1.921		(1.141, 3.235)			0.014		1.225		(0.836, 1.794)		0.298		1.337		(0.928, 1.926)		0.119		1.882		(1.047, 3.386)		0.035		1.093		(0.754, 1.584)		0.638
Flowers with pollen (yes)														1.203		(0.802, 1.804)		0.371		1.552		(0.810, 2.975)		0.185		1.204		(0.800, 1.812)		0.372
Using insecticide (yes)																				0.440		(0.241, 0.806)		0.008						

^a^All models were adjusted for age of children, children gender (male/female), family history of asthma (yes/no), tenure status (owner/tenant), and smoking people in family (yes/no).

^b^Final model of each symptom was performed using selected variables which had significant value less than 0.2 (p-value < 0.2) in bivariate analysis.

## Discussion

Respiratory and asthma symptoms in the past 12 months among primary school children in the highly polluted area were reported at around 1 in 4. Running nose without cold was reported in the highest numbers, and shortness of breath was reported in the lowest numbers. Wall dampness, present of vectors, living near garment and clothing shops were indicated to be risk factors for respiratory and asthma symptoms among the primary school children in this study, while age and gender of children could modify the associations.

Our study evaluated the association between residential environment and respiratory symptoms in an urban area of Bangkok, where the concentration of air pollution was the highest. We found that around 11% of the children in our study had wheezing symptoms. The prevalence of respiratory symptoms in our study was lower than a study of Liu et al.^15^ in urban Shanghai which was 21.7% and a study of Mathew et al.^16^ in Delhi which found rank between 12.7–17.7%. In addition, a majority of reported respiratory symptoms in our study was dry cough at night (32.5%) and phlegm (43.3%) which was accordance with a study of Mathew et al.^16^ in Delhi.

Home dampness-related exposure is a risk for childhood respiratory diseases^17^. In our study, present of wall dampness in the residence was associated with parent reported asthma and shortness of breath among their children. Our finding was consistent with several studies^10,18^. Dampness is known as a cause of mold growth which was a trigger allergen^19^. Sun et al.^17^ conducted a study between the dampness-exposure indices and childhood respiratory and allergic diseases and found that the dampness indices had exposure–response relationships with childhood respiratory and allergic diseases. The possible mechanism is that microbes and mycotoxins produced irritative and volatile organic compounds which may induce IgE-mediated hypersensitivity of the respiratory tract^11^.

Residential proximity to a source of air pollution including garment and clothing factory was associated with shortness of breath among children in our finding. However, the proximity to source of pollution was a parent reported. Garment and clothing factory in the city can be a source of air pollutants including microplastics (MPs). The synthetic fabric from clothing is recognize as a source of MPs besided tire erosion (especially from automobiles and trucks), household objects, waste incineration, and building materials^20^. A garment may release approximately 1900 fibres per wash^21^. Beside, cutting and grinding processes for polymeric materials can contribute to the formation and release of these particles in the air^22^. Therefore, living near the garment factory in inner city may unintentionally expose to the MPs. The relationship between MPs exposure and respiratory effects is still unclear. The possible mechanism is that fibres can be deposited in terminal bronchioles, alveolar ducts, and alveoli which are contributed to chronic inflammation, granulomas or fibrosis^23^.

Exposure to vectors including cockroach and rat in the house increased the odds of the symptoms of a dry cough at night (p < 0.05) and phlegm (p < 0.05) among the children. Vectors were recognized as risk factors for allergy symptoms and respiratory symptoms, including asthma in children. The presence of cockroaches in indoor dirt and decay in the residence was the key allergens in inner-city homes^24^. A possible mechanism could be cockroaches contain proteolytic digestive enzymes, which directly induce bronchial inflammation^25^ and associated with cockroach-specific IgE which is a critical factor in lowering pulmonary function^26^. The worst asthma cases were related to high concentrations of cockroach allergens in the residence, and the tendency to allergic reactions to cockroach allergens, which increased the severity of asthma^27^.

Type of residence and house owner status are the possible predictors of health status among children. Majority of children were living in flats, apartments, and condominiums with the length of residence was more than 30 years. Most of parent also reported that their house was tenant. The results could suggested that being house owners take better care of their houses compared with tenants. Moreover, house ownership may be related to better health outcomes because it could indicate higher income, wealth, better housing infrastructure, and healthier neighborhood conditions.

The present study has several limitations. First, a self-reported questionnaire was used as the measurement tool, which may have led to information bias. Moreover, parents who live in worse residential environments may be more likely to overreport respiratory symptoms^28^. In a future study, hospital-based records of respiratory and asthma disease should be considered. Asthma status and symptoms should be validated by physician diagnosis, medications, or emergency department visits to minimize self-reporting biases. Second, only two primary schools in the Din Daneng district, which is controlled by the Bangkok Metropolitan Administration, were selected. Therefore, the findings of this study cannot be generalized to other primary school children in urban areas. However, to the best of our knowledge, this is the first study to investigate the relationship between home environments and respiratory symptoms among a large sample size of primary school children in a highly polluted area in Bangkok, Thailand. Third, the respiratory and asthma symptoms in this study were considered over a long term (12 months), which may have led to recall bias. Fourth, our study did not collect samples of residential and school indoor air quality to confirm an association between air quality and health outcomes. However, our study included all significant predictors in residential environments of respiratory health in children. Finally, our study did not consider the sizes or processes of businesses near the children’s residences, including furniture shops, garment/clothing factories, and garage/car care facilities. Differences in factory processes may lead to different emissions. A future study should investigate the emissions from each type of factory and business.

The findings of our study showed that residential environments, including tenant status, garment/clothing shops near residence, cigarette smoke, and incense smoke were positively and significantly associated with respiratory and asthma symptoms. Further interventions to improve residential environments and control housing quality should be considered to reduce respiratory and asthma symptoms among children in urban areas.

## Methods

### Study participants and study area

A cross-sectional study was conducted during the summer of 2018. The Din Daeng district was selected as the study setting because it is the highest polluted area in inner Bangkok, according to the Pollution Control Department (PCD) of Thailand. As discussed in “Excessive PM2.5 in 69 areas of Bangkok” (2020), the PCD reported that a 24-h mean of particulate matter of less than 2.5 µm (PM2.5) in Din Daeng exceeded the Thailand air quality standard in 27% of all measurements, while in the Thonburi district it exceeded 11% of all measurements.

Two of three primary public schools in the district took part in this study. All children in two primary public schools were invited to participate. Of the total 814 students, 658 primary school children who met the inclusion criteria were willing to be included in the study. The inclusion criteria were children aged between 6 and 10 years who had lived in the Din Daeng district for at least one year. Students who were absent on the day of recruitment were asked to participate in the study on the day that they were present. This study was approved by the Ethics Review Committee of Chulalongkorn University (COA No. 085/2561). This research was performed in accordance with relevant guidelines and regulations. Informed consent was obtained for all parents of the participants.

### Questionnaire

The self-reported questionnaire was a translated to Thai language form of the International Study of Asthma and Allergies in Childhood (ISAAC) questionnaire. The translation and back-translation were done to achieve a validity of questionnaire. The parents of the children provided information about residential environments and respiratory and asthma symptoms in their child during the previous 12 months, such as wheezing or whistling in the chest (i.e., asthma), dry cough at night, phlegm, shortness of breath, and running nose without cold symptoms. Based on the World Allergy Organization’s criteria for diagnosing asthma symptoms in children, a positive answer to the question, “Have your child had those symptoms in the past 12 months?” was considered to indicate respiratory and asthma symptoms. A positive response to questions about having experienced wheezing or whistling in the chest during the past 12 months was also considered to indicate asthma^29^.

The children’s characteristics and residential environmental conditions were reported by their parents. The characteristics were include;

**Table:** 

Age of the child	Year
Gender	Male/female
Height	Centimeter (cm)
Weight at present	Kilograms (kg)
Family history of asthma	Yes/no
Exercise	Yes/no

The residential conditions included;

**Table:** 

Type of residence	Single-family house/townhouse/flat/apartment/condominium/slum
Age of residence	Years
Ownership status of residents	Owner/tenant
Places near the residence (less than 100 m)	Furniture shop (yes/no)
	Garment and/or clothing shop (yes/no)
	Garage (yes/no)
	Car care (yes/no)
	Petrol station (yes/no)
	Fresh market and/or restaurant (cooking smoke) (yes/no)
Family member smoker	Yes/no
Stove used for cooking	Yes/no
Presence of wall dampness during the last 12 months	Yes/no
Home renovation during the last 12 months	Yes/no
Insecticide used during the last 12 month	Yes/no
Presence of flowers with pollen	Yes/no
Presence of vectors during the last 12 month	Yes/no
Pets	Yes/no
Child living in incense smoke area	Yes/no
Child living in cigarette smoke area	Yes/no

### Statistical analysis

SPSS version 22 was used to conduct all statistical analyses in this study. Regarding descriptive statistics, categorical data were reported by frequency and percentage. Continuous data were presented as mean and standard deviation (SD). Cases of skewed, median, and interquartile ranking (IQR) were reported. The Chi-square and Fisher’s exact tests were applied to assess the association among categorical data in the bivariate analysis. Continuous variables were evaluated using the Mann–Whitney *U* test to observe differences between having and not having respiratory and asthma symptoms. A multivariate analysis was performed using a binary logistic regression. In each respiratory symptom, the final model included variables that showed significant values of less than 0.2 in the bivariate analysis (p < 0.20). The potential confounders of age, gender (male/female), family history of asthma (yes/no), ownership status of residents (owner/tenant), and smokers among family members (yes/no) were included in the models in an a priori approach. Adjusted odd ratios (AOR) with 95% confidence intervals were presented. All reported p-values were two-sided and defined at a 5% level of significance. The final models were evaluated for sensitivity analysis by excluding 2.3% of the children who had a family history of asthma (Supplement Table S3). Effect modification was explored by age group (younger group, 6–7 years; older, 8–10 years) (Supplement Table S4) and sex (Supplement Table S5).

## Supplementary Information

Supplementary Table S1.

Supplementary Table S2.

Supplementary Table S3.

Supplementary Table S4.

Supplementary Table S5.
