# Supplementary material for: Residential environment in relation to self-report of respiratory and asthma symptoms among primary school children in a high-polluted urban area
Source: Sci Rep. 2022 Feb 22;12:2946. doi: 10.1038/s41598-022-06919-9 (PMC8863880; doi:10.1038/s41598-022-06919-9)
Supplement: Supplementary file 1 — Supplementary Table S1. [file 41598_2022_6919_MOESM1_ESM.docx]

**Table S1** Prevalence of respiratory and asthma symptoms

| **Symptoms** | Yes: n (%) | | No: n (%) | | |  |
| --- | --- | --- | --- | --- | --- | --- |
| Wheezing or whistling in the chest (Asthma) | 75 | (11.4) | | 583 | (88.6) | |
| Dry cough at night | 214 | (32.5) | | 444 | (67.5) | |
| Phlegm | 285 | (43.3) | | 373 | (56.7) | |
| Shortness of breath | 60 | (9.1) | | 598 | (90.9) | |
| Running nose without cold | 347 | (52.7) | | 311 | (47.3) | |
