# Supplementary material for: Residential environment in relation to self-report of respiratory and asthma symptoms among primary school children in a high-polluted urban area
Source: Sci Rep. 2022 Feb 22;12:2946. doi: 10.1038/s41598-022-06919-9 (PMC8863880; doi:10.1038/s41598-022-06919-9)
Supplement: Supplementary file 2 — Supplementary Table S2. [file 41598_2022_6919_MOESM2_ESM.docx]

**Table S2** Residential environment and respiratory/ asthma symptoms of primary school children (n=658)

|  | **Total  (n=658)** | | **Wheezing or whistling  in the chest (Asthma)** | | | | | | | | **Dry cough at night** | | | | | | | | **Phlegm** | | | | | | | | |  |
| --- | --- | --- | --- | --- | --- | --- | --- | --- | --- | --- | --- | --- | --- | --- | --- | --- | --- | --- | --- | --- | --- | --- | --- | --- | --- | --- | --- | --- |
| **Residential environment** | n (%) | | Yes (n=75): n (%) | | | No (n=583): n (%) | | | p-value | | Yes (n=214): n (%) | | | No (n=444) : n (%) | | | p-value | | | Yes (n=285): n (%) | | | No (n=373): n (%) | | | p-value | | |
| Family member; Median (IQR) | 3 | (1.0) | 3 | (2.0) | 3 | | (1.0) | 0.456*^a^* | | 3 | | (2.0) | 3 | | (1.0) | 0.150*^a^* | | 3 | | | (2.0) | 3 | | (1.0) | 0.543*^a^* | |  |  |
| Living in cigarette smoke area |  |  |  |  |  | |  | 0.631*^b^* | |  | |  |  | |  | 0.005*^b^* | |  | | |  |  | |  | 0.092*^b^* | |  |  |
| Yes | 85 | (12.9) | 11 | (12.9) | 74 | | (87.1) |  | | 39 | | (45.9) | 46 | | (54.1) |  | | 44 | | | (51.8) | 41 | | (48.2) |  | |  |  |
| No | 573 | (87.1) | 64 | (11.2) | 509 | | (88.8) |  | | 175 | | (30.5) | 398 | | (69.5) |  | | 241 | | | (42.1) | 332 | | (57.9) |  | |  |  |
| Have smoking people in family |  |  |  |  |  | |  | 0.914*^b^* | |  | |  |  | |  | 0.024*^b^* | |  | | |  |  | |  | 0.062*^b^* | |  |  |
| Yes | 312 | (47.4) | 36 | (11.5) | 276 | | (88.5) |  | | 115 | | (36.9) | 197 | | (63.1) |  | | 147 | | | (47.1) | 165 | | (52.9) |  | |  |  |
| No | 346 | (52.6) | 39 | (11.3) | 307 | | (88.7) |  | | 99 | | (28.6) | 247 | | (71.4) |  | | 138 | | | (39.9) | 208 | | (60.1) |  | |  |  |
| Using stove (for cooking) |  |  |  |  |  | |  | 0.718*^b^* | |  | |  |  | |  | 0.567*^b^* | |  | | |  |  | |  | 0.682*^b^* | |  |  |
| Yes | 106 | (16.1) | 11 | (10.4) | 95 | | (89.6) |  | | 37 | | (34.9) | 69 | | (65.1) |  | | 44 | | | (41.5) | 62 | | (58.5) |  | |  |  |
| No | 552 | 83.9 | 64 | (11.6) | 488 | | (88.4) |  | | 177 | | (32.1) | 375 | | (67.9) |  | | 241 | | | (43.7) | 311 | | (56.3) |  | |  |  |
| Charcoal smoke (Cooking stove) |  |  |  |  |  | |  | 0.344*^b^* | |  | |  |  | |  | 0.742*^b^* | |  | | |  |  | |  | 0.425*^b^* | |  |  |
| Yes | 84 | (12.8) | 7 | (8.3) | 77 | | (91.7) |  | | 26 | | (31.0) | 58 | | (69.0) |  | | 33 | | | (39.3) | 51 | | (60.7) |  | |  |  |
| No | 574 | (87.2) | 68 | (11.8) | 506 | | (88.2) |  | | 188 | | (32.8) | 386 | | (67.2) |  | | 252 | | | (43.9) | 322 | | (56.1) |  | |  |  |
| Living in charcoal smoke area |  |  |  |  |  | |  | 1.000*^c^* | |  | |  |  | |  | 0.646*^b^* | |  | | |  |  | |  | 0.567*^b^* | |  |  |
| Yes | 41 | (6.2) | 4 | (9.8) | 37 | | (90.2) |  | | 12 | | (29.3) | 29 | | (70.7) |  | | 16 | | | (39.0) | 25 | | (61.0) |  | |  |  |
| No | 617 | (93.8) | 71 | (11.5) | 546 | | (88.5) |  | | 202 | | (32.7) | 415 | | (67.3) |  | | 269 | | | (43.6) | 348 | | (56.4) |  | |  |  |
| Incense use |  |  |  |  |  | |  | 0.375*^b^* | |  | |  |  | |  | 0.473*^b^* | |  | | |  |  | |  | 0.612*^b^* | |  |  |
| Yes | 140 | (21.3) | 13 | (9.3) | 127 | | (90.7) |  | | 42 | | (30.0) | 98 | | (70.0) |  | | 58 | | | (41.4) | 82 | | (58.6) |  | |  |  |
| No | 518 | (78.7) | 62 | (12.0) | 456 | | (88.0) |  | | 172 | | (33.2) | 346 | | (66.8) |  | | 227 | | | (43.8) | 291 | | (56.2) |  | |  |  |
| Living in incense smoke area |  |  |  |  |  | |  | 0.178*^c^* | |  | |  |  | |  | 0.330*^b^* | |  | | |  |  | |  | 0.274*^b^* | |  |  |
| Yes | 24 | (3.6) | 5 | (20.8) | 19 | | (79.2) |  | | 10 | | (41.7) | 14 | | (58.3) |  | | 13 | | | (54.2) | 11 | | (45.8) |  | |  |  |
| No | 634 | (96.4) | 70 | (11.0) | 564 | | (89.0) |  | | 204 | | (67.8) | 430 | | (32.2) |  | | 272 | | | (42.9) | 362 | | (57.1) |  | |  |  |
| Wall dampness |  |  |  |  |  | |  | 0.007*^b^* | |  | |  |  | |  | 0.083*^b^* | |  | | |  |  | |  | 0.024*^b^* | |  |  |
| Yes | 163 | (24.8) | 28 | (17.2) | 135 | | (82.8) |  | | 62 | | (38.0) | 101 | | (62.0) |  | | 83 | | | (50.9) | 80 | | (49.1) |  | |  |  |
| No | 495 | (75.2) | 47 | (9.5) | 448 | | (90.5) |  | | 152 | | (30.7) | 343 | | (69.3) |  | | 202 | | | (40.8) | 293 | | (59.2) |  | |  |  |
| Home renovation |  |  |  |  |  | |  | 0.515*^b^* | |  | |  |  | |  | 0.029*^b^* | |  | | |  |  | |  | 0.028*^b^* | |  |  |
| Yes | 142 | (21.6) | 14 | (9.9) | 128 | | (90.1) |  | | 57 | | (40.1) | 85 | | (59.9) |  | | 73 | | | (51.4) | 69 | | (48.6) |  | |  |  |
| No | 516 | (78.4) | 61 | (11.8) | 455 | | (88.2) |  | | 157 | | (30.4) | 359 | | (69.6) |  | | 212 | | | (41.1) | 304 | | (58.9) |  | |  |  |
| Using insecticide |  |  |  |  |  | |  | 0.844*^b^* | |  | |  |  | |  | 0.481*^b^* | |  | | |  |  | |  | 0.860*^b^* | |  |  |
| Yes | 494 | (75.1) | 57 | (11.5) | 437 | | (88.5) |  | | 157 | | (31.8) | 337 | | (68.2) |  | | 213 | | | (43.1) | 281 | | (56.9) |  | |  |  |
| No | 164 | (24.9) | 18 | (11.0) | 146 | | (89.0) |  | | 57 | | (34.8) | 107 | | (65.2) |  | | 72 | | | (43.9) | 92 | | (56.1) |  | |  |  |
| Flowers with pollen |  |  |  |  |  | |  | 0.758*^b^* | |  | |  |  | |  | 0.670*^b^* | |  | | |  |  | |  | 0.175*^b^* | |  |  |
| Yes | 123 | (18.7) | 15 | (12.2) | 108 | | (87.8) |  | | 42 | | (34.1) | 81 | | (65.9) |  | | 60 | | | (48.8) | 63 | | (51.2) |  | |  |  |
| No | 535 | (81.3) | 60 | (11.2) | 475 | | (88.8) |  | | 172 | | (32.1) | 363 | | (67.9) |  | | 225 | | | (57.9) | 310 | | (42.1) |  | |  |  |
| Vectors (cockroach, rat, etc.) |  |  |  |  |  | |  | 0.192*^b^* | |  | |  |  | |  | 0.005*^b^* | |  | | |  |  | |  | 0.006*^b^* | |  |  |
| Yes | 411 | (62.5) | 52 | (12.7) | 359 | | (87.3) |  | | 150 | | (36.5) | 261 | | (63.5) |  | | 195 | | | (47.4) | 216 | | (52.6) |  | |  |  |
| No | 247 | (37.5) | 23 | (9.3 | 224 | | (90.7) |  | | 64 | | (25.9) | 183 | | (74.1) |  | | 90 | | | (36.4) | 157 | | (63.6) |  | |  |  |
| Pets (dog, cat, bird, etc.) |  |  |  |  |  | |  | 0.531*^b^* | |  | |  |  | |  | 0.253*^b^* | |  | | |  |  | |  | 0.143*^b^* | |  |  |
| Yes | 311 | (47.3) | 38 | (12.2) | 273 | | (87.8) |  | | 108 | | (34.7) | 203 | | (65.3) |  | | 144 | | | (46.3) | 167 | | (53.7) |  | |  |  |
| No | 347 | (52.7) | 37 | (10.7) | 310 | | (89.3) |  | | 106 | | (30.5) | 241 | | (69.5) |  | | 141 | | | (40.6) | 206 | | (59.4) |  | |  |  |

*Note. ^a^* Mann -Whitney U test, *^b^* Pearson Chi-Square test

**Table S2** Residential environment and respiratory/ asthma symptoms of primary school children (n=658) (Continued)

|  | **Total  (n=658)** | | **Shortness of breath** | | | | | **Running nose without cold** | | | | |
| --- | --- | --- | --- | --- | --- | --- | --- | --- | --- | --- | --- | --- |
| **Residential environment** | n (%) | | Yes (n=60):  n (%) | | No (n=598): n (%) | | p-value | Yes (n=347): n (%) | | No (n=311): n (%) | | p-value |
| Family member; Median (IQR) | 3 | (1.0) | 3 | (2.0) | 3 | (2.0) | 0.187*^a^* | 3 | (2.0) | 3 | (1.0) | 0.456*^a^* |
| Living in cigarette smoke area (Yes) |  |  |  |  |  |  | 0.364*^b^* |  |  |  |  | 0.095*^b^* |
| Yes | 85 | (12.9) | 10 | (11.8) | 75 | (88.2) |  | 52 | (61.2) | 33 | (38.8) |  |
| No | 573 | (87.1) | 50 | (8.7) | 523 | (91.3) |  | 295 | (51.5) | 278 | (48.5) |  |
| Have smoking people in family (Yes) |  |  |  |  |  |  | 0.903*^b^* |  |  |  |  | 0.004*^b^* |
| Yes | 312 | (47.4) | 28 | (9.0) | 284 | (91.0) |  | 183 | (58.7) | 129 | (41.3) |  |
| No | 346 | (52.6) | 32 | (9.2) | 314 | (90.8) |  | 164 | (47.4) | 182 | (52.6) |  |
| Using stove (for cooking) (Yes) |  |  |  |  |  |  | 0.902*^b^* |  |  |  |  | 0.656*^b^* |
| Yes | 106 | (16.1) | 10 | (9.4) | 96 | (90.6) |  | 58 | (54.7) | 48 | (45.3) |  |
| No | 552 | 83.9 | 50 | (9.1) | 502 | (90.9) |  | 289 | (52.4) | 263 | (47.6) |  |
| Charcoal smoke (Cooking stove) (Yes) |  |  |  |  |  |  | 0.501*^b^* |  |  |  |  | 0.944*^b^* |
| Yes | 84 | (12.8) | 6 | (7.1) | 78 | (92.9) |  | 44 | (52.4) | 40 | (47.6) |  |
| No | 574 | (87.2) | 54 | (9.4) | 520 | (90.6) |  | 303 | (52.8) | 271 | (47.2) |  |
| Living in charcoal smoke area (Yes) |  |  |  |  |  |  | 0.254*^c^* |  |  |  |  | 0.841*^b^* |
| Yes | 41 | (6.2) | 6 | (14.6) | 35 | (85.4) |  | 21 | (51.2) | 20 | (48.8) |  |
| No | 617 | (93.8) | 54 | (8.8) | 563 | (91.2) |  | 326 | (52.8) | 291 | (47.2) |  |
| Incense use (Yes) |  |  |  |  |  |  | 0.460*^b^* |  |  |  |  | 0.727*^b^* |
| Yes | 140 | (21.3) | 15 | (10.7) | 125 | (89.3) |  | 72 | (51.4) | 68 | (48.6) |  |
| No | 518 | (78.7) | 45 | (8.7) | 473 | (91.3) |  | 275 | (53.1) | 243 | (46.9) |  |
| Living in incense smoke area (Yes) |  |  |  |  |  |  | 0.058*^c^* |  |  |  |  | 0.070*^b^* |
| Yes | 24 | (3.6) | 5 | (20.8) | 19 | (79.2) |  | 17 | (70.8) | 7 | (29.2) |  |
| No | 634 | (96.4) | 55 | (8.7) | 579 | (91.3) |  | 330 | (52.1) | 304 | (47.9) |  |
| Wall dampness (Yes) |  |  |  |  |  |  | 0.004*^b^* |  |  |  |  | 0.146*^b^* |
| Yes | 163 | (24.8) | 24 | (14.7) | 139 | (85.3) |  | 94 | (57.7) | 69 | (42.3) |  |
| No | 495 | (75.2) | 36 | (7.3) | 459 | (92.7) |  | 253 | (51.1) | 242 | (48.9) |  |
| Home renovation (Yes) |  |  |  |  |  |  | 0.096*^b^* |  |  |  |  | 0.021*^b^* |
| Yes | 142 | (21.6) | 18 | (12.7) | 124 | (87.3) |  | 87 | (61.3) | 55 | (38.7) |  |
| No | 516 | (78.4) | 42 | (8.1) | 474 | (91.9) |  | 260 | (50.4) | 256 | (49.6) |  |
| Using insecticide (Yes) |  |  |  |  |  |  | 0.058*^b^* |  |  |  |  | 0.788*^b^* |
| Yes | 494 | (75.1) | 39 | (7.9) | 455 | (92.1) |  | 262 | (53.0) | 232 | (47.0) |  |
| No | 164 | (24.9) | 21 | (12.8) | 143 | (87.2) |  | 85 | (51.8) | 79 | (48.2) |  |
| Flowers with pollen (Yes) |  |  |  |  |  |  | 0.097*^b^* |  |  |  |  | 0.153*^b^* |
| Yes | 123 | (18.7) | 16 | (13.0) | 107 | (87.0) |  | 72 | (58.5) | 51 | (41.5) |  |
| No | 535 | (81.3) | 44 | (8.2) | 491 | (91.8) |  | 275 | (51.4) | 260 | (48.6) |  |
| Vectors (cockroach, rat, etc.) (Yes) |  |  |  |  |  |  | 0.122*^b^* |  |  |  |  | 0.009*^b^* |
| Yes | 411 | (62.5) | 43 | (10.5) | 368 | (89.5) |  | 233 | (56.7) | 178 | (43.3) |  |
| No | 247 | (37.5) | 17 | (6.9) | 230 | (93.1) |  | 114 | (46.2) | 133 | (53.8) |  |
| Pets (dog, cat, bird, etc.) (Yes) |  |  |  |  |  |  | 0.656*^b^* |  |  |  |  | 0.435*^b^* |
| Yes | 311 | (47.3) | 30 | (9.6) | 281 | (90.4) |  | 169 | (54.3) | 142 | (45.7) |  |
| No | 347 | (52.7) | 30 | (8.6) | 317 | (91.4) |  | 178 | (51.3) | 169 | (48.7) |  |

*Note. ^a^* Mann -Whitney U test, *^b^* Pearson Chi-Square test
