# Supplementary material for: Residential environment in relation to self-report of respiratory and asthma symptoms among primary school children in a high-polluted urban area
Source: Sci Rep. 2022 Feb 22;12:2946. doi: 10.1038/s41598-022-06919-9 (PMC8863880; doi:10.1038/s41598-022-06919-9)
Supplement: Supplementary file 3 — Supplementary Table S3. [file 41598_2022_6919_MOESM3_ESM.docx]

**Table S3** Binary logistic regression model of associations between residential environment and respiratory/ asthma symptoms of children without a family history of asthma (n=643)

|  | Wheezing or whistling  in the chest (Asthma) | | | | Dry cough at night | | | | Phlegm | | | | Shortness of breath | | | | Running nose without cold | | | |
| --- | --- | --- | --- | --- | --- | --- | --- | --- | --- | --- | --- | --- | --- | --- | --- | --- | --- | --- | --- | --- |
| **Factors** | AOR (95% CI) | | | *p*-value | AOR (95% CI) | | | *p*-value | AOR (95% CI) | | | *p*-value | AOR (95% CI) | | | *p*-value | AOR (95% CI) | | | *p*-value |
| Age of residence (year) |  |  |  |  | 1.009 | (0.994, | 1.023) | 0.239 | 1.007 | (0.994, | 1.020) | 0.297 |  |  |  |  |  |  |  |  |
| Place near residence |  |  |  |  |  |  |  |  |  |  |  |  |  |  |  |  |  |  |  |  |
| Garment/ clothing (Yes) |  |  |  |  |  |  |  |  |  |  |  |  | **1.824** | **(1.009** | **3.297)** | **0.047** |  |  |  |  |
| Furniture shop (Yes) |  |  |  |  |  |  |  |  |  |  |  |  | 1.794 | (0.673, | 4.781) | 0.242 |  |  |  |  |
| Garage/ car care (Yes) | 0.508 | (0.174, | 1.479) | 0.214 |  |  |  |  |  |  |  |  |  |  |  |  |  |  |  |  |
| Fresh market and restaurant (cooking smoke) (Yes) |  |  |  |  |  |  |  |  |  |  |  |  |  |  |  |  | 0.211 | (0.057, | 0.782) | 0.020 |
| Living in cigarette smoke area (Yes) |  |  |  |  | **1.723** | **(1.020,** | **2.909)** | **0.042** | 1.228 | (0.735, | 2.051) | 0.432 |  |  |  |  | 1.072 | (0.629, | 1.825) | 0.799 |
| Living in incense smoke area (Yes) | 2.201 | (0.755, | 6.415) | 0.148 |  |  |  |  |  |  |  |  | 2.986 | (0.966, | 9.234) | 0.058 | 1.813 | (0.701, | 4.685) | 0.220 |
| Vectors (cockroach, rat, etc.) (Yes) | 1.303 | (0.757, | 2.240) | 0.339 | **1.592** | **(1.106,** | **2.293)** | **0.012** | 1.364 | (0.974, | 1.912) | 0.071 | 1.283 | (0.688, | 2.390) | 0.433 | 1.348 | (0.966, | 1.881) | 0.079 |
| Home renovation (Yes) |  |  |  |  | 1.397 | (0.923, | 2.116) | 0.114 | 1.341 | (0.899, | 2.001) | 0.151 | 1.672 | (0.887, | 3.152) | 0.112 | 1.483 | (0.985, | 2.231) | 0.059 |
| Wall dampness (Yes) | **1.811** | **(1.060,** | **3.094)** | **0.030** | 1.117 | (0.753, | 1.657) | 0.581 | 1.303 | (0.897, | 1.897) | 0.165 | 1.793 | (0.978, | 3.290) | 0.059 | 1.075 | (0.735, | 1.572) | 0.708 |
| Flowers with pollen (Yes) |  |  |  |  |  |  |  |  | 1.200 | (0.795, | 1.810) | 0.386 | 1.672 | (0.863, | 3.238) | 0.128 | 1.208 | (0.797, | 1.832) | 0.373 |
| Using insecticide (Yes) |  |  |  |  |  |  |  |  |  |  |  |  | 0.489 | (0.261, | 0.914) | 0.025 |  |  |  |  |

*^a^All models were adjusted for age of children, children gender (male/female), tenure status (owner/ tenant), and smoking people in family (yes/no)*
