# Supplementary material for: Residential environment in relation to self-report of respiratory and asthma symptoms among primary school children in a high-polluted urban area
Source: Sci Rep. 2022 Feb 22;12:2946. doi: 10.1038/s41598-022-06919-9 (PMC8863880; doi:10.1038/s41598-022-06919-9)
Supplement: Supplementary file 4 — Supplementary Table S4. [file 41598_2022_6919_MOESM4_ESM.docx]

**Table S4** Binary logistic regression model of associations between residential environment and respiratory/ asthma symptoms of children stratified by age group

|  | Wheezing or whistling  in the chest (Asthma) | | | | Dry cough at night | | | | Phlegm | | | | Shortness of breath | | | | Running nose without cold | | | |
| --- | --- | --- | --- | --- | --- | --- | --- | --- | --- | --- | --- | --- | --- | --- | --- | --- | --- | --- | --- | --- |
| **Factors** | AOR (95% CI) | | | *p*-value | AOR (95% CI) | | | *p*-value | AOR (95% CI) | | | *p*-value | AOR (95% CI) | | | *p*-value | AOR (95% CI) | | | *p*-value |
| **Younger children (age 6 – 7 years; n=216)** | | | | | | | | | | | | | | | | | | | | |
| Age of residence (year) |  |  |  |  | 1.003 | (0.976, | 1.023) | 0.842 | 0.995 | (0.969, | 1.021) | 0.736 |  |  |  |  |  |  |  |  |
| Place near residence |  |  |  |  |  |  |  |  |  |  |  |  |  |  |  |  |  |  |  |  |
| Garment/ clothing (Yes) |  |  |  |  |  |  |  |  |  |  |  |  | **3.971** | **(1.414,** | **11.15)** | **0.009** |  |  |  |  |
| Furniture shop (Yes) |  |  |  |  |  |  |  |  |  |  |  |  | 4.993 | (0.886, | 28.11) | 0.068 |  |  |  |  |
| Garage/ car care (Yes) | 0.559 | (0.063, | 4.941) | 0.559 |  |  |  |  |  |  |  |  |  |  |  |  |  |  |  |  |
| Fresh market and restaurant (cooking smoke) (Yes) |  |  |  |  |  |  |  |  |  |  |  |  |  |  |  |  |  |  |  |  |
| Living in cigarette smoke area (Yes) |  |  |  |  | 1.343 | (0.524, | 3.439) | 0.538 | 1.391 | (0.547, | 3.535) | 0.488 |  |  |  |  | 1.745 | (0.621, | 4.903) | 0.291 |
| Living in incense smoke area (Yes) | 4.955 | (0.733, | 33.51) | 0.101 |  |  |  |  |  |  |  |  | 4.919 | (0.759, | 31.87) | 0.095 | 0.816 | (0.171, | 3.897) | 0.799 |
| Vectors (cockroach, rat, etc.) (Yes) | 0.541 | (0.194, | 1.507) | 0.541 | 0.829 | (0.455, | 1.509) | 0.540 | 1.338 | (0.749, | 2.391) | 0.325 | 0.467 | (0.158, | 1.380) | 0.169 | 1.094 | (0.613, | 1.950) | 0.761 |
| Home renovation (Yes) |  |  |  |  | 1.571 | (0.778, | 3.171) | 0.207 | 0.820 | (0.407, | 1.649) | 0.578 | 2.377 | (0. 770, | 7.337) | 0.132 | 1.204 | (0.596, | 2.434) | 0.605 |
| Wall dampness (Yes) | 2.525 | (0.845, | 7.544) | 0.097 | 1.398 | (0.694, | 2.812) | 0.348 | 1.836 | (0.932, | 3.618) | 0.079 | **3.342** | **(1.133,** | **9.859)** | **0.029** | 1.810 | (0.889, | 3.685) | 0.102 |
| Flowers with pollen (Yes) |  |  |  |  |  |  |  |  | 0.507 | (0.227, | 1.129) | 0.097 | 0.684 | (0.219, | 2.129) | 0.128 | 0.668 | (0.309, | 1.446) | 0.306 |
| Using insecticide (Yes) |  |  |  |  |  |  |  |  |  |  |  |  | 0.489 | (0.261, | 0.914) | 0.512 |  |  |  |  |
| **Older children (age 8-10 years; n= 442)** | | | | | | | | | | | | | | | | | | | | |
| Age of residence (year) |  |  |  |  | 1.012 | (0.996, | 1.029) | 0.138 | 1.014 | (0.998, | 1.030) | 0.069 |  |  |  |  |  |  |  |  |
| Place near residence |  |  |  |  |  |  |  |  |  |  |  |  |  |  |  |  |  |  |  |  |
| Garment/ clothing (Yes) |  |  |  |  |  |  |  |  |  |  |  |  | 1.428 | (0.664, | 3.067) | 0.362 |  |  |  |  |
| Furniture shop (Yes) |  |  |  |  |  |  |  |  |  |  |  |  | 0.846 | (0.219, | 3.276) | 0.809 |  |  |  |  |
| Garage/ car care (Yes) | 0.448 | (0.132, | 1.528) | 0.200 |  |  |  |  |  |  |  |  |  |  |  |  |  |  |  |  |
| Fresh market and restaurant (cooking smoke) (Yes) |  |  |  |  |  |  |  |  |  |  |  |  |  |  |  |  | 0.305 | (0.090, | 1.028) | 0.055 |
| Living in cigarette smoke area (Yes) |  |  |  |  | 1.759 | (0.933, | 3.319) | 0.081 | 1.193 | (0.638, | 2.233) | 0.579 |  |  |  |  | 0.870 | (0.460, | 1.644) | 0.667 |
| Living in incense smoke area (Yes) | 1.724 | (0.449, | 6.611) | 0.427 |  |  |  |  |  |  |  |  | 2.005 | (0.398, | 10.10) | 0.399 | 2.536 | (0.747, | 8.603) | 0.136 |
| Vectors (cockroach, rat, etc.) (Yes) | 1.744 | (0.895, | 3.397) | 0.102 | **2.127** | **(1.333,** | **3.395)** | **0.002** | 1.490 | (0.980, | 2.267) | 0.062 | 2.740 | (1.116, | 6.725) | 0.028 | 1.564 | (1.037, | 2.361) | 0.033 |
| Home renovation (Yes) |  |  |  |  | 1.189 | (0.714, | 1.980) | 0.503 | 1.593 | (0.977, | 2.599) | 0.062 | 1.285 | (0.566, | 2.915) | 0.549 | 1.492 | (0.908, | 2.454) | 0.115 |
| Wall dampness (Yes) | 1.648 | (0.893, | 3.042) | 0.110 | 1.162 | (0.726, | 1.858) | 0.531 | 1.083 | (0.689, | 1.701) | 0.730 | 1.367 | (0.641, | 2.915) | 0.419 | 0.862 | (0.548, | 1.356) | 0.520 |
| Flowers with pollen (Yes) |  |  |  |  |  |  |  |  | 1.680 | (1.023, | 2.759) | 0.040 | 2.058 | (0.905, | 4.680) | 0.085 | 1.513 | (0.917, | 2.498) | 0.105 |
| Using insecticide (Yes) |  |  |  |  |  |  |  |  |  |  |  |  | 0.294 | (0.134, | 0.644) | 0.002 |  |  |  |  |

*^a^All models were adjusted for children gender (male/female), family history of asthma (yes/no), tenure status (owner/ tenant), and smoking people in family (yes/no)*
