# Supplementary material for: Residential environment in relation to self-report of respiratory and asthma symptoms among primary school children in a high-polluted urban area
Source: Sci Rep. 2022 Feb 22;12:2946. doi: 10.1038/s41598-022-06919-9 (PMC8863880; doi:10.1038/s41598-022-06919-9)
Supplement: Supplementary file 5 — Supplementary Table S5. [file 41598_2022_6919_MOESM5_ESM.docx]

**Table S5** Binary logistic regression model of associations between residential environment and respiratory/ asthma symptoms of children stratified by gender

|  | Wheezing or whistling  in the chest (Asthma) | | | | | | Dry cough at night | | | | | | | | Phlegm | | | | | | | | Shortness of breath | | | | | | | | Running nose without cold | | | | |
| --- | --- | --- | --- | --- | --- | --- | --- | --- | --- | --- | --- | --- | --- | --- | --- | --- | --- | --- | --- | --- | --- | --- | --- | --- | --- | --- | --- | --- | --- | --- | --- | --- | --- | --- | --- |
| **Factors** | AOR (95% CI) | | | | *p*-value | | AOR (95% CI) | | | | | | *p*-value | | AOR (95% CI) | | | | | | *p*-value | | AOR (95% CI) | | | | | | *p*-value | | AOR (95% CI) | | | | *p*-value |
| **Male (n= 330)** | | | | | | | | | | | | | | | | | | | | | | | | | | | | | | | | | | | |
| Age of residence (year) |  |  | |  |  | | 1.016 | | (0.994, | | 1.037) | | 0.139 | | 1.011 | | (0.991, | | 1.031) | | 0.271 | |  | |  | |  | |  | |  | |  |  |  |
| Place near residence |  |  | |  |  | |  | |  | |  | |  | |  | |  | |  | |  | |  | |  | |  | |  | |  | |  |  |  |
| Garment/ clothing (Yes) |  |  | |  |  | |  | |  | |  | |  | |  | |  | |  | |  | | 1.265 | | (0.514, | | 3.117) | | 0.608 | |  | |  |  |  |
| Furniture shop (Yes) |  |  | |  |  | |  | |  | |  | |  | |  | |  | |  | |  | | 1.551 | | (0.370, | | 6.492) | | 0.547 | |  | |  |  |  |
| Garage/ car care (Yes) | 0.946 | (0.302, | | 2.958) | 0.924 | |  | |  | |  | |  | |  | |  | |  | |  | |  | |  | |  | |  | |  | |  |  |  |
| Fresh market and restaurant (cooking smoke) (Yes) |  |  | |  |  | |  | |  | |  | |  | |  | |  | |  | |  | |  | |  | |  | |  | | **0.061** | | **(0.005,** | **0.690)** | **0.024** |
| Living in cigarette smoke area (Yes) |  |  | |  |  | | **2.857** | | **(1.313,** | | **6.218)** | | **0.008** | | 1.540 | | (0.723, | | 3.280) | | 0.262 | |  | |  | |  | |  | | 1.808 | | (0.785, | 4.166) | 0.164 |
| Living in incense smoke area (Yes) | **5.683** | **(1.413,** | | **22.85)** | **0.014** | |  | |  | |  | |  | |  | |  | |  | |  | | **8.409** | | **(1.667,** | | **42.42)** | | **0.010** | | **23.33** | | **(1.653,** | **329.2)** | **0.020** |
| Vectors (cockroach, rat, etc.) (Yes) | 0.937 | (0.471, | | 1.861) | 0.853 | | 1.564 | | (0.939, | | 2.606) | | 0.085 | | 1.030 | | (0.640, | | 1.658) | | 0.903 | | 1.361 | | (0.566, | | 3.275) | | 0.491 | | 1.082 | | (0.672, | 1.741) | 0.746 |
| Home renovation (Yes) |  |  | |  |  | | 1.467 | | (0.826, | | 2.605) | | 0.190 | | **1.888** | | **(1.088,** | | **3.277)** | | **0.024** | | **2.370** | | **(1.006,** | | **5.585)** | | **0.049** | | **1.866** | | **(1.046,** | **3.331)** | **0.035** |
| Wall dampness (Yes) | 1.261 | (0.606, | | 2.627) | 0.534 | | 1.153 | | (0.669, | | 1.986) | | 0.608 | | 1.256 | | (0.751, | | 2.101) | | 0.384 | | 1.932 | | (0.826, | | 4.520) | | 0.129 | | 0.914 | | (0.540, | 1.550) | 0.740 |
| Flowers with pollen (Yes) |  |  | |  |  | |  | |  | |  | |  | | 0.988 | | (0.530, | | 1.841) | | 0.970 | | 1.286 | | (0.441, | | 3.749) | | 0.645 | | 1.056 | | (0.561, | 1.988) | 0.866 |
| Using insecticide (Yes) |  |  | |  |  | |  | |  | |  | |  | |  | |  | |  | |  | | **0.401** | | **(0.167,** | | **0.960)** | | **0.040** | |  | |  |  |  |
| **Female (n= 328)** | | | | | | | | | | | | | | | | | | | | | | | | | | | | | | | | | | | |
| Age of residence (year) |  |  | |  |  | | 1.002 | | (0.983, | | 1.021) | | 0.783 | | 1.006 | | (0.988, | | 1.025) | | 0.503 | |  | |  | |  | |  | |  | |  |  |  |
| Place near residence |  |  | |  |  | |  | |  | |  | |  | |  | |  | |  | |  | |  | |  | |  | |  | |  | |  |  |  |
| Garment/ clothing (Yes) |  |  | |  |  | |  | |  | |  | |  | |  | |  | |  | |  | | 1.174 | | (0.515, | | 2.678) | | 0.703 | |  | |  |  |  |
| Furniture shop (Yes) |  |  | |  |  | |  | |  | |  | |  | |  | |  | |  | |  | | **2.996** | | **(1.333,** | | **6.733)** | | **0.008** | |  | |  |  |  |
| Garage/ car care (Yes) |  |  |  | | |  | |  | |  | |  | |  | |  | |  | |  | |  | |  | |  | |  | |  | |  |  |  |  |
| Fresh market and restaurant (cooking smoke) (Yes) |  |  | |  |  | |  | |  | |  | |  | |  | |  | |  | |  | |  | |  | |  | |  | | 0.382 | | (0.074, | 1.978) | 0.251 |
| Living in cigarette smoke area (Yes) |  |  | |  |  | | 1.117 | | (0.531, | | 2.350) | | 0.769 | | 1.036 | | (0.504, | | 2.130) | | 0.922 | |  | |  | |  | |  | | 0.827 | | (0.392, | 1.746) | 0.619 |
| Living in incense smoke area (Yes) | 0.761 | (0.083, | | 6.898) | 0.808 | |  | |  | |  | |  | |  | |  | |  | |  | | 2.468 | | (0.595, | | 10.22) | | 0.213 | | 0.494 | | (0.135, | 1.811) | 0.287 |
| Vectors (cockroach, rat, etc.) (Yes) | 2.085 | (0. 796, | | 5.464) | 0.135 | | 1.333 | | (0.792, | | 2.243) | | 0.279 | | **1.751** | | **(1.077,** | | **2.846)** | | **0.024** | | 1.938 | | (0.313, | | 12.00) | | 0.477 | | 1.589 | | (0.980, | 2.576) | 0.060 |
| Home renovation (Yes) |  |  | |  |  | | 1.018 | | (0.547, | | 1.896) | | 0.955 | | 0.804 | | (0.442, | | 1.463) | | 0.476 | | 1.175 | | (0.471, | | 2.926) | | 0.730 | | 1.050 | | (0.575, | 1.918) | 0.875 |
| Wall dampness (Yes) | **3.479** | **(1.573,** | | **7.697)** | **0.002** | | 1.376 | | (0.782, | | 2.420) | | 0.268 | | 1.421 | | (0.819, | | 2.466) | | 0.211 | | 0.801 | | (0.277, | | 2.321) | | 0.683 | | 1.446 | | (0.814, | 2.570) | 0.209 |
| Flowers with pollen (Yes) |  |  | |  |  | |  | |  | |  | |  | | 1.333 | | (0.760, | | 2.339) | | 0.316 | | 2.118 | | (0.878, | | 5.109) | | 0.095 | | 1.260 | | (0.710, | 2.234) | 0.430 |
| Using insecticide (Yes) |  |  | |  |  | |  | |  | |  | |  | |  | |  | |  | |  | | 1.769 | | (0.743 | | 4.213) | | 0.198 | |  | |  |  |  |

*^a^All models were adjusted for age of children(years), family history of asthma (yes/no), tenure status (owner/ tenant), and smoking people in family (yes/no)*
